# Integrated multisystem analysis in a mental health and criminal justice ecosystem

**DOI:** 10.1186/s40352-017-0049-y

**Published:** 2017-03-22

**Authors:** Erin Falconer, Tal El-Hay, Dimitris Alevras, John P Docherty, Chen Yanover, Alan Kalton, Yaara Goldschmidt, Michal Rosen-Zvi

**Affiliations:** 1 ODH, Inc., 508 Carnegie Center, Princeton, NJ 08540 USA; 2 IBM Research, IBM R&D Labs in Israel, Haifa University Campus, Mount Carmel, Haifa, 3498825 Israel; 3 IBM Global Business Services, 1475 Phoenixville Pike, West Chester, PA 19380 USA; 4 IBM Research, Catholic University of Eastern Africa, P.O Box 62157 Bogani E Rd, Nairobi, Kenya; 5 Present Address: Ernst & Young, 1 Maagplatz, 8005 Zürich, Switzerland

**Keywords:** Serious mental illness, Criminal justice system, Arrest, Healthcare system, Hospitalization, Risk factors

## Abstract

**Background:**

Patients with a serious mental illness often receive care that is fragmented due to reduced availability of or access to resources, and inadequate, discontinuous, and uncoordinated care across health, social services, and criminal justice organizations. This article describes the creation of a multisystem analysis that derives insights from an integrated dataset including patient access to case management services, medical services, and interactions with the criminal justice system.

**Methods:**

Data were combined from electronic systems within a US mental health ecosystem that included mental health and substance abuse services, as well as data from the criminal justice system. Cox models were applied to test the associations between delivery of services and re-incarceration. Additionally, machine learning was used to train and validate a predictive model to examine effects of non-modifiable risk factors (age, past arrests, mental health diagnosis) and modifiable risk factors (outpatient, medical and case management services, and use of a jail diversion program) on re-arrest outcome.

**Results:**

An association was found between past arrests and admission to crisis stabilization services in this population (*N* = 10,307). Delivery of case management or medical services provided after release from jail was associated with a reduced risk for re-arrest. Predictive models linked non-modifiable and modifiable risk factors and outcomes and predicted the probability of re-arrests with fair accuracy (area under the receiver operating characteristic curve of 0.67).

**Conclusions:**

By modeling the complex interactions between risk factors, service delivery, and outcomes, systems of care might be better enabled to meet patient needs and improve outcomes.

**Electronic supplementary material:**

The online version of this article (doi:10.1186/s40352-017-0049-y) contains supplementary material, which is available to authorized users.

## Background

The mental healthcare system in the United States is fragmented, inconsistent, and underfunded. Dominated by a lack of consistency and care continuity, the system allows many individuals with mental illness to go untreated, remain unstable, and/or to mentally decompensate, leading to crisis outcomes such as homelessness, hospitalizations, and imprisonment. Fragmentation of care for supporting individuals with mental illness is particularly evidenced by the observation that in the prison population the proportion of individuals with serious mental illness is larger than that found in the general population; it has been estimated that 15 to 25% of the adult prison population suffers from serious mental illness (Dickson et al. 2006; James and Glaze 2006; Torrey et al. 2010), versus an estimated prevalence of 5 to 8% found in the general population (Substance Abuse and Mental Health Services Administration 2014). One study of state and federal prisons and local jails estimated that 45 to 64% of inmates in the United States have a recent history or symptoms of a mental health problem, including 15 to 24% of state jail inmates reporting symptoms meeting the criteria for a psychotic disorder (James and Glaze 2006). Many individuals with severe mental illness are released from prison in the United States and re-enter the community with a need for mental health treatment, which could help prevent relapse and recidivism.

The lack of continuous care for adults with serious mental illness who are navigating the mental health, social, and criminal justice systems also limits our ability to perform research to determine ways in which we might intervene to address the risk factors for adverse outcomes. With fragmented, discontinuous care, and different agencies maintaining isolated datasets, there is a lack of access to continuous patient-level data. This makes it difficult to collate data across health, social, and criminal justice agencies and to evaluate the interplay between risk factors, the delivery of services, and outcomes. A critical need exists to evaluate continuous patient-level and service-level data across multiple agencies in order to understand the mechanisms through which we may intervene to prevent or delay psychiatric crisis.

Previous work evaluating data from US Medicaid claim files and arrest records found a reduced risk of re-arrest with receipt of outpatient services (Gilbert et al. 2010; Morrissey et al. 2007; Van Dorn et al. 2013) and psychotropic medication possession (Van Dorn et al. 2013) in adults with mental illness. Other research using county- and statewide criminal justice records and archival data from health and social services found that individual risk factors including being homeless, not having outpatient mental health treatment, and having involuntary psychiatric evaluation in the previous quarter, and being black, younger than 21 years and having a co-occurring substance abuse problem increased the odds of arrest (Constantine et al. 2010). Recent studies for other medical applications have used electronic medical records data to establish predictive models for illness severity in various disease domains, including preterm infants (Saria et al. 2010), congestive heart failure (Sun et al. 2012), septic shock (Paxton et al. 2013) and HIV (Zazzi et al. 2011).

In this retrospective study, we describe an approach to modeling the interplay among services and outcomes across a state-funded ecosystem of medical and social services providers of care for mental illness or substance abuse, and the criminal justice system. We explored associations between the occurrence of arrest and modifiable and non-modifiable risk factors tested using hazard modeling with both fixed- and time-dependent covariates. Finally, we demonstrate the utility of such a combined dataset for predicting re-arrest.

## Methods

### Framework

This study uses information extracted from electronic systems resident within a healthcare ecosystem in the southern US which funds the care of a state-funded, indigent population of individuals with mental illness and/or substance abuse issues. The included data were obtained from the claims process for medical and social services delivered to mental health and substance abuse patients across a network of publically-funded care providers, as well as data from the criminal justice system database. All individual patient data used for the analysis were collected by providers after obtaining appropriate consents and agreements. A third party processed the provider data to remove any personal information to protect patient privacy. The resulting de-identified data sets were then made available for analysis.

Patient data collected for this analysis span 21 months and describe the engagements that patients have with service providers in the ecosystem. In the longitudinal data we defined an index date, where data collected before that date serve as input and data after that time point define outcome values. We then define the target populations and an outcome measure. Finally, we extract and filter features and risk factors to drive the modeling (Additional file 1: Figure S1).

### End points

The main outcome measure was re-arrest in individuals who had become involved in the criminal justice system (Additional file 1: Figure S1). The study explored two main questions: what are the modifiable and non-modifiable risk factors associated with re-arrest, and how well can we predict the likelihood of re-arrest using such risk factors. Our approach employed an association analysis and a machine learning analysis to explore the answers to the first and second questions, respectively. Additionally, we tested the association of risk factors to crisis stabilization unit (CSU) admission. The CSU in this system is a state-supported mental health service, providing brief and intensive services for those who are suffering from acute mental illness, and functioning as a short-term alternative to inpatient psychiatric hospitalization. The CSU examines, stabilizes, and refers individuals to the most appropriate treatment settings.

We hypothesized that the relationship between mental health services and the criminal justice system may be bidirectional; thus, we tested associations between risk factors including previous arrest records and the risk of admission to acute mental health treatment.

### Data sources

Datasets for mental health and substance abuse admissions (points of service entry) and events were included which span October 1, 2010 to June 30, 2012 and were obtained for the indigent population across a network of publically funded mental healthcare and substance abuse providers. All mental health admissions before October 1, 2010 were recorded with an admission date of October 1, 2010. Substance abuse admissions data cover July 1, 2009 and June 30, 2012. We focused on individuals with serious mental illness (SMI) who had one of the following diagnoses: bipolar disorder (International Classification of Diseases, 9th edition [ICD-9] codes: 296 to 296.19 and 296.40 to 296.99); schizophrenic disorder (ICD-9 codes: 295 to 295.99 and 297 to 298.99); major depression (ICD-9 codes: 296.20 to 296.39).

From the datasets for mental health and substance abuse admissions and events, the cohort included a population of 29,558 individuals with a SMI diagnosis of bipolar disorder, schizophrenic disorder, and/or major depression.

Arrest data were supplied by the Department of Law Enforcement and extracted from the Criminal Justice Information Services (CJIS) spanning a period from January 1, 2007 to September 6, 2012, and included records on 184,470 individuals. Of these 184,470 individuals, 5148 overlap with the SMI population in the health ecosystem studied. The court provided a list of participants in a program called the Jail Diversion Program (JDP), overlapping with the population contained in the other data sources. The JDP seeks to reintroduce individuals with mental illness into a sustained care environment, combining mental health and housing services as part of a structured year-long engagement. Participation in the JDP was used to confirm the set of specific services provided to individuals selected for analysis (*n* = 812), and also as one of the informative covariates in the predictive modeling.

### Population selection

To analyze the relations between arrest and behavioral health service events we focused on a subset of the adult population having records both in the CJIS and mental health ecosystem datasets (*n* = 5148). We excluded 281 individuals from this cohort because of inconsistent timeline data (such as release without arrest, admission or events with unrealistic dates). Of the remaining individuals (*n* = 4867), a total of 3274 were released from an arrest after October 1, 2010, which is the starting date of the mental health services recorded in the dataset. Of these, 3171 were adults at the time of release (we included individuals with estimated age >18 years at release).

**Fig. 1:**
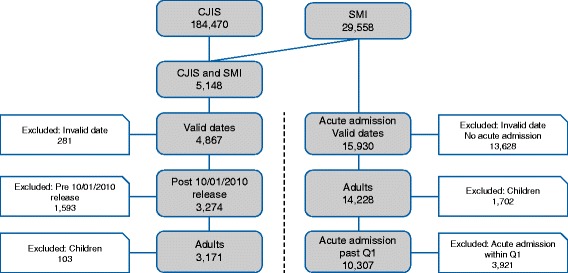
Selection of patient populations. CJIS = Criminal Justice Information Services; SMI = serious mental illness

We analyzed the association of past arrests with admission to acute mental health treatment using the SMI cohort, excluding individuals whose first recorded admission had ambiguous or unknown dates, creating a subset of 15,930 individuals. Of these, we focused on the adult population (*N* = 14,228). Selection of the 2 populations (re-arrest model and acute treatment model) is shown in Fig. 1.

### Statistical analysis

#### Association analysis using Cox models

Cox proportional hazard models were used for testing associations between risk factors and expected time for failure events to occur (Cox 1972). This association is modeled using a hazard rate representing the amount of risk as a function of time. The effect of each risk factor is assumed to be multiplicative with respect to the hazard rate. Past arrest factors were modeled as indicator variables whose value was one if the individual was arrested and released from jail between January 2007 (start date of the CJIS data) and October 2010, and zero otherwise.

We predicted the effect of services given immediately after release and, additionally, examined the effect of continuous access to services. These tests involve time dependent covariates such as access to services in every month after release from jail. Association tests with such covariates were performed using extended Cox models (see Pettitt and Daud (Pettitt and Daud 1990) for more details).

#### Predictive modeling using elastic nets

An elastic net method allows classical regression models to deal with high dimensionality of observations. This method performs data-driven variable selection and results in a sparse model that includes the most informative covariates. Learning in such models involves tuning of two parameters: alpha, which controls the sparseness and stability of the model, where a higher alpha increases the tendency of the learning algorithm to filter out non-informative covariates; beta, a regularization parameter that prevents over-fitting of the model to the data which is employed to obtain optimal generalization performance. These parameters are usually tuned using internal cross-validation on a training data set. The accuracy of the model is assessed on a test set. For more details, see Zou and Hastie (Zou and Hastie 2005).

### Analyses

#### Association between non-modifiable risk factors, receiving services after release from jail, and the risk of re-arrest

The initial association analysis examined non-modifiable risk factors including gender, age, race, mental health diagnosis, and past arrests using a Cox proportional hazard model. The association of receiving different service types with the risk of re-arrest was evaluated, adjusting for these non-modifiable risk factors. The dataset contains 39 types of services (Additional file 2: Table S1), of which 14 were provided to >20 patients in the cohort. Each of these service types was represented using an indicator covariate equal to one if an individual received the service at least once in the first quarter after release from jail and equal to zero otherwise.

#### Continuous access to services

Extended Cox models were used to examine the association of services given throughout the entire period after release from jail to the risk of re-arrest. For each patient, all release dates after Oct 1, 2010 were listed and corresponding re-arrest dates were identified (or, if the patient was not re-arrested, the end-of-study date was used). Starting from each such release date, the number of times each service was given to the patient in each consecutive 90-day period was tabulated.

Subsets of these time-varying covariates, in addition to the non-modifiable factors, were then used to infer the parameters of extended Cox models. Models were constructed with indicator covariates identifying a service given within the last 90 days or since the last release from jail to predict re-arrest within the coming 90 days.

#### Predictive modeling using elastic nets

To test the predictability of the arrest outcome, data were partitioned into a training set containing approximately 80% of the cohort and a test set containing the remaining 20%. Because the goal of the analysis was to predict probability of re-arrest in the second quarter after release, the target population was similar to the one described in re-arrest risk factor analysis although individuals for whom 2 quarters of data were not available were excluded. The training set thus comprised 1679 individuals and the test set 421. We evaluated the predictive power of an elastic net regularized regression model using a receiver operating characteristic (ROC) curve, which compares the likelihood of correctly and incorrectly predicting re-arrest.

## Results

### Preliminary associations of demographic and historical factors with re-arrest

Demographic characteristics of the 2 populations are shown in Table 1. Preliminary associations (i.e., without adjusting for other variables) between non-modifiable risk factors and the risks of re-arrest are summarized in Table 2. In particular, schizophrenia, history of arrests, male gender, black race, and younger age were shown to be risk factors for increased likelihood of re-arrest.

**Table 1:** Demographic characteristics

	Re-arrest		Acute treatment	
	(*N* = 3171)		(*N* = 14228)	
Characteristic	N	%	N	%
Gender				
Male	2244	70.8	6921	48.6
Female	927	29.2	7305	51.3
Race				
Black	1376	43.4	3740	26.3
White	483	15.2	2672	18.8
Hispanic	1272	40.1	7579	53.3
Other/unknown	40	1.3	237	1.7
Diagnosis				
Bipolar	836	26.4	3133	22
Schizophrenia	1592	50.2	5314	37.3
Major Depression	743	23.4	5781	40.6
Age, mean (SD)	38.0 (12.4)		44.5 (14.9)	
Past arrests, mean (SD)	0.6 (0.5)		0.1 (0.2)

**Table 2:** Preliminary associations between baseline characteristics and the risk of re-arrest

Factor	Crude hazard ratio	95% confidence interval	*P*-value
Gender (Female vs. Male)	0.71	0.63–0.81	<0.001
Race (Black vs. Other)	1.31	1.18–1.47	<0.001
Diagnosis (vs. Major Depression)			
Bipolar disorder	1.22	1.03–1.45	0.02
Schizophrenic disorders	1.50	1.30–1.74	<0.001
Past arrests	2.04	1.80–2.31	<0.001
Age	.99	0.99–1.00	<0.001

### Association between arrests and crisis services

In the ecosystem studied, a large proportion of individuals were first admitted into the system of care through a CSU (representing more than 30% within a week from first admission). A subset of the population was examined that did not record an admission to a CSU in the first quarter (*N* = 10,307). In this sample, the association of a later CSU admission with past arrests, adjusted for age, gender, race, and mental health diagnosis was significant (*p* < 0.001) with a high hazard ratio (HR) of 2.46 (95% confidence interval 2.00–3.02).

### Services associated with reduced risk of re-arrest

To test associations with services given in the first quarter after release, inmates that were re-arrested within the quarter were excluded. Of 3171 adults, 2377 (~75%) remained out of jail during this period. This test therefore was able to examine conditional probabilities of future re-arrests given that the individual remained out of jail in the first quarter.

**Fig. 2:**
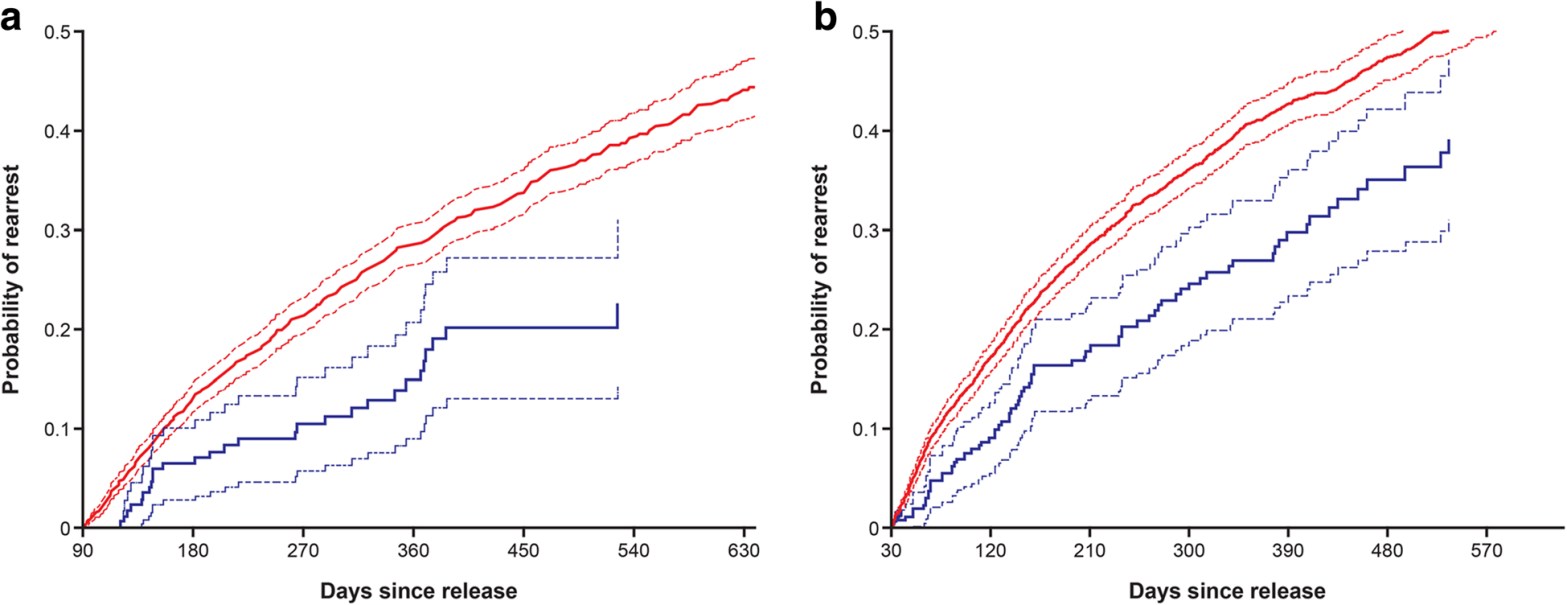
Effect of case management (**a**) and access to medical services (**b**) on arrest probability. Kaplan-Meier estimators. Red line=did not receive service, blue line=received service

Associations between service indicator variables and the risk of re-arrest adjusting for gender, age, race, mental health diagnosis and past arrests as defined in the baseline test model were also tested. Overall, we tested a few hundred such associations, with service indicators computed over different time windows following release from jail and, therefore, consider associations with *p* < 0.0001 significant at a .05 level after the Bonferroni correction. Results, summarized in Table 3, indicate an association of case management and medical services with a reduced risk of re-arrest. Figure 2 presents Kaplan-Meier plots of arrest probability for individuals who stayed out of jail in the first quarter after release, given their access to these services in this quarter.

**Table 3:** Associations among services and the risk of re-arrest^a^

Factor	N	Adjusted hazard ratio	95% confidence interval	*P*-value
Case management	172	0.45	0.30–0.68	<0.001
Medical services	491	0.59	0.47–0.74	<0.001
Outpatient group^b^	45	0.46	0.22–0.96	0.038

^a^The CJIS data include both booking and arrest records. The table above refers to booking dates. Using arrest dates instead, gives a 0.48 hazard ratio for case management and 0.64 for medical services

^b^This association is not considered significant as it does not pass a multiple hypothesis correction

### Continuous access to care and continuous monitoring of patients’ states

Extended Cox model analysis shows that the indicator for Medical Services, either in the past 90 days or since release, is significantly associated with a reduced risk for re-arrest, with HRs of .68 (confidence interval 0.58–0.80, *p* < 0.0015) and .67 (.58–.78, *p* < 0.001), respectively. Conversely, the indicator for Crisis Stabilization, in both time periods, is associated with elevated levels of re-arrest, with HRs of 1.43 (confidence interval 1.22–1.69, *p* < 0.001) and 1.23 (1.07–1.42, *p* = 0.003), respectively. Schonenfeld residuals for all these indicators, except for Crisis Stabilization in the past 90 days, attested to the correctness of the proportional hazard assumption.

### Predictive modeling of re-arrests

**Fig. 3:**
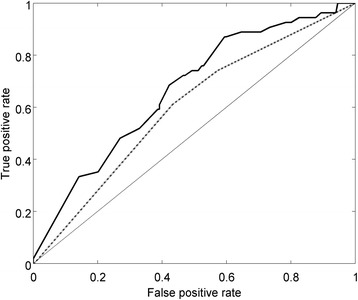
ROC curve of elastic net predictive model for re-arrest outcome. ROC = receiver operating characteristic. Basic model=dotted line; Full model=solid line

Elastic net regularized logistic regression models were trained using a training set containing 1679 individuals. The regularization parameters alpha and beta were tuned using cross validation and the model was retrained on the entire training set with optimal parameters. Testing this model on a test set of 421 individuals resulted in an area under the receiver operating characteristic curve (AUC) of 0.67 (see ‘Full model’ in Fig. 3). Informative covariates selected by the training procedure included age, past arrests, mental health diagnosis, enrollment to the JDP, as well as utilization of outpatient group services, medical services and case management. The probability of re-arrest is modeled as function of a weighted sum of these factors. As the ROC curve in Fig. 3 indicates, the model correctly predicts 50% of individuals in the ecosystem at risk for re-arrest based on the defined risk factors, while mischaracterizing 30% of individuals at risk. To assess the predictability of re-arrest from basic demographic data, namely, age, gender and race, we trained a simpler model using the same cohort and an elastic net model. This model was inferior to the full model, with an AUC of 0.60 and 42% true positive rate at the 30% false positive threshold (‘Basic model’ in Fig. 3). The difference between the two ROCs illustrates the additional predictive power of the judicial and mental health related factors.

## Discussion

In this analysis, we found that characteristics including schizophrenia, history of arrests, male gender, black race, and younger age were risk factors for increased likelihood of re-arrest. A history of arrests was positively associated with admission to crisis stabilization, whereas receipt of case management services or medical services following release from jail was associated with a reduced likelihood of re-arrest. Using these key modifiable and non-modifiable risk factors, we created a model which predicted the probability of re-arrest after the first 90 days of release from jail with fair accuracy.

Our findings showing a greater risk for arrest for individuals who are young, male and of black race support previous work that examined US Medicaid claims and arrests records, and other sources, and found that minority racial-ethnic status (Van Dorn et al. 2013) or African American race (Constantine et al. 2010; Gilbert et al. 2010), male gender (Gilbert et al. 2010), and younger age (Gilbert et al. 2010) are associated with arrest.

After adjusting for gender, age, race, mental health diagnosis and indicator of past arrests, we tested whether receipt of healthcare services within the first 3 months after release predicted risk for future re-arrest in those individuals who were able to stay out of jail (75% of our cohort remained out of jail during the subsequent quarter). We found that the receipt of case management services predicted an over 50% reduction in risk for re-arrest, and a little over 40% reduction in risk for those who received medical services. The receipt of other behavioral health services, such as outpatient group therapy, was not considered significant. This is in-line with previous findings that have shown that the delivery of outpatient services, which may have included individual or group behavioral health services, medication that checks (Morrissey et al. 2007), and/or case management (Gilbert et al. 2010; Van Dorn et al. 2013), predict reductions in recidivism in the mentally ill. However, the current findings extend this previous work by considering the independent contributions of case management and medical services in predicting re-arrest risk in those with serious mental illness. In the behavioral healthcare ecosystem studied, case management included assessment, coordination of services, referral and follow-up of clinical services. Medical services included primary medical care, psychiatric assessment, therapy and medication administration. The relative contribution of each of these elements of care and/or assessment services within the broader categories of “medical services” and “care management” to the findings is unclear. For example, it may be that receipt of medication following arrest contributed to a reduction of risk, given that medication possession 90 days after hospitalization reduces the risk of arrest (Van Dorn et al. 2013). Our dataset did not allow us to do an analysis of the effect of the specific elements, or subsets of services, within each service category, and their associations with re-arrest risk. This is a limitation of the current dataset and analyses, and is therefore an avenue for further research.

Our finding that arrests were positively associated with utilization of CSU services suggests a situation in which individuals who are arrested may also be those who are suffering the most from acute mental illness and need acute care. The relationship found between CSU utilization and arrests does not infer causality; instead, our findings may reflect that relatively less stable individuals are at greater risk for both arrest and mental health crisis. This is aligned with suggestions that jails may now be considered the “new mental health hospitals” (Johnson et al. 2015; Torrey et al. 2010), given research showing that there are significantly greater odds that an individual with serious mental illness will end up in prison than in hospital care (Johnson et al. 2015; Torrey et al. 2010). Our analyses do not infer causal relationships, but, taken together with the finding that those receiving case management and medical services are less likely to be re-arrested, our analysis at the very least provides support for the suggestion that mental health stability in a population at risk for arrest is related to a reduction in risk for subsequent re-arrest. This has implications for mental health policy and practice; it indicates the importance of prioritizing mental health care in populations at risk for reincarceration, and finding new ways in which we can coordinate the delivery of mental health services to those interacting with the criminal justice system.

Understanding how we can best intervene to stabilize mental health and multiple incarcerations for individuals with mental health issues may be important not only for improving the quality of life of adults with SMI, but also for reducing costs within the systems that support their care. Using Florida Medicaid data and records from Florida’s Department of Children and Families and the Florida Department of Law Enforcement, Van Dorn et al (Van Dorn et al. 2013) compared the costs associated with criminal justice system involvement with those for mental health treatment, and found that overall system costs were lower for adults with SMI who were not arrested. Taken together with our current findings, the results suggest that increasing the provision of case management and medical services in a SMI population at risk for arrest may be an important strategy for reducing overall system cost burden. This should be explored in future research.

In the current analysis, we chose to include individuals with depression, bipolar disorder, and schizophrenia because these diagnoses and related symptoms are found at a high rate in the criminal justice system (Ditton 1999; James and Glaze 2006). Future work should explore the role of other mental health diagnoses in predictive models, as well as the effect of comorbid conditions. Future studies should also be conducted to refine the model by integrating other sources of data (e.g. additional medical claims, pharmacy and hospitalization data). In the current analysis, medical services included primary medical care, psychiatric assessment and services, and administration of psychotropic drugs and other medications. It would be of particular interest for future analyses to examine the specific role of pharmacy/medication administration in predicting re-arrest, particularly given recent data showing that post-hospitalization medication possession reduced the likelihood of arrest in adults with SMI in a Florida Medicaid population (Van Dorn et al. 2013).

The focus on an indigent care population represents a limitation to the results of the study. Given the nature of the health issues involved, and the impact of poor mental health on social integration and economic status, it is a relevant segment to focus on for the analysis. However, it would be interesting for further analysis to explore and contrast experience with a non-indigent population. Potential access to different services, support structures and different quality and connectivity of services could result in different findings.

## Conclusions

Our findings illustrate the complex interactions between modifiable and non-modifiable risk factors and delivery of services on outcomes in adults with SMI. The data-driven approach defined in this analysis demonstrates the value of integrating data across disparate datasets from healthcare, social services, and criminal justice agencies. Further development of this predictive model may help identify those individuals at greater risk for re-arrest and crisis, and to intervene in a timely manner to help improve outcomes for the mentally ill. A reduction in arrests in this seriously mentally ill population may not only improve patient outcomes, but also diminish the burden on the judicial and health systems.

## Additional files

Additional file 1: Figure S1. Data preprocessing and feature extraction framework. (TIFF 32 kb)

Additional file 2: Table S1. Different types of mental health services provided to individuals after release from jail. (DOCX 35 kb)

## Abbreviations

AUC: Area under the concentration versus time curve
CJIS: Criminal Justice Information Services
CSU: Crisis stabilization unit
ICD-9: International Classification of Diseases, 9th edition
JDP: Jail Diversion Program
ROC: Receiver operating characteristic
SMI: Serious mental illness
