# Supplementary figures and images for: Integrated multisystem analysis in a mental health and criminal justice ecosystem
Source: Health Justice. 2017 Mar 22;5:4. doi: 10.1186/s40352-017-0049-y (PMC5362563; doi:10.1186/s40352-017-0049-y)

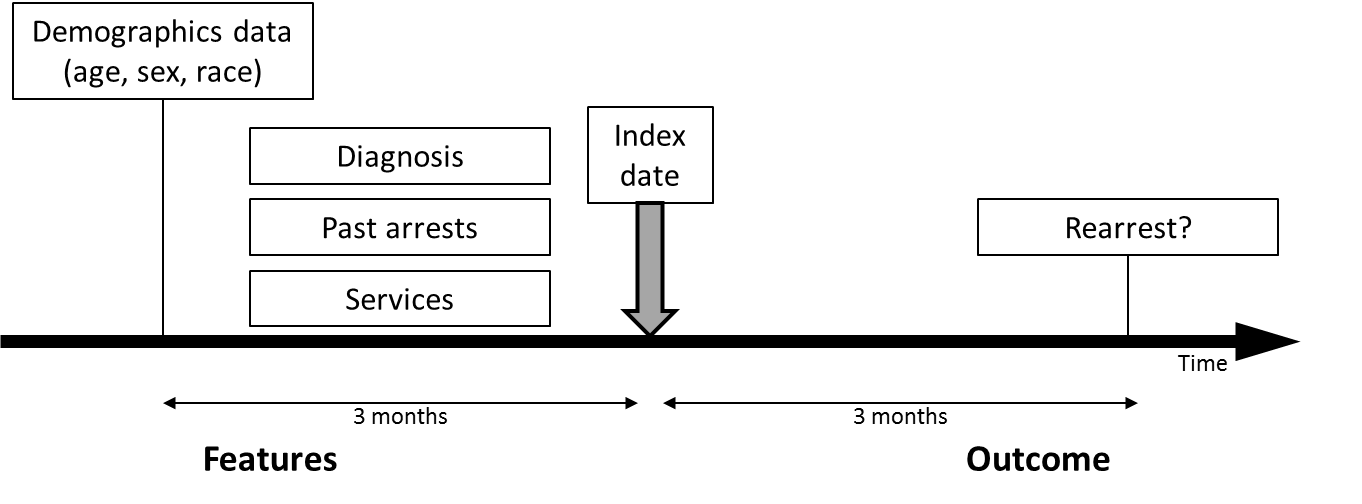

Supplement: Supplementary file 1 — Data preprocessing and feature extraction framework. (TIFF 32 kb) [file 40352_2017_49_MOESM1_ESM.tiff]
