# Supplementary material for: Integrated multisystem analysis in a mental health and criminal justice ecosystem
Source: Health Justice. 2017 Mar 22;5:4. doi: 10.1186/s40352-017-0049-y (PMC5362563; doi:10.1186/s40352-017-0049-y)
Supplement: Supplementary file 2 — Different types of mental health services provided to individuals after release from jail. (DOCX 35 kb) [file 40352_2017_49_MOESM2_ESM.docx]

**Supplemental Table. Different Types of Mental Health Services Provided to Individuals After Release From Jail**

| **Service** | **Definition** |
| --- | --- |
| Assessment | Assessment services assess, evaluate, and provide assistance to individuals and families to determine level of care, motivation, and the need for services and supports to assist individuals and families identify their strengths. |
| Case Management | Case management services consist of activities aimed at identifying the recipient's needs, planning services, linking the service system with the person, coordinating the various system components, monitoring service delivery and evaluating the effect of the services received. |
| Crisis Stabilization | These acute care services provide, on a twenty-four (24) hours per day, seven ( 7) days per week basis, provide brief, intensive mental health residential treatment services to meet the needs of individuals who are experiencing an acute crisis and who, in the absence of a suitable alternative, would require hospitalization. |
| Crisis Support/Emergency | These non-residential care services are generally available twenty-four (24) hours per day, seven (7) days per week, or some other specific time period, to intervene in a crisis or provide emergency care. Examples include: mobile crisis, crisis support, crisis/emergency screening, crisis telephone and emergency walk-in. |
| Day/Night | Day-night services provide a structured schedule of non-residential services for four (4) or more consecutive hours per day. Activities for children and adult mental health programs are designed to assist individuals to attain skills and behaviors needed to function successfully in living, learning, work, and social environments. Generally, a person receives three (3) or more services a week. Activities for substance abuse programs emphasize rehabilitation, treatment, and education services, using multidisciplinary teams to provide integrated programs of academic, therapeutic, and family services. |
| In-Home & On-Site Services | Therapeutic services and supports are rendered in non-provider settings such as nursing homes, alternative living facilities, residences, schools, detention centers, commitment settings, foster homes, and other community settings. |
| Intensive Case Management | Case management services consist of activities aimed at assessing recipient needs, planning services, linking the service system to a recipient, coordinating the various system components, monitoring service delivery and evaluating the effect of services received. These services are typically offered to persons who are being discharged from a hospital or crisis stabilization unit, who are in need of more professional care, and who will have contingency needs to remain in a less restrictive setting. |
| Intervention | Intervention services focus on reducing risk factors generally associated with the progression of substance abuse and mental health problems. Intervention is accomplished through early identification of persons at risk, performing basic individual assessments, and providing supportive services, which emphasize short-term counseling and referral. These services are targeted toward individuals and families. |
| Medical Services | Medical services provide primary medical care, therapy and medication administration to improve the functioning or prevent further deterioration of persons with mental health or substance abuse problems. Included is psychiatric mental status assessment.  For adults with mental illness, medical services are usually provided on a regular schedule, with arrangements for non-scheduled visits during times of increased stress or crisis. This service includes medication administration of psychotropic drugs including Clozaril and other new medications, and psychiatric services. |
| Methadone Maintenance | Methadone medication maintenance consist of a group of outpatient services, which utilize methadone and other opioid replacement therapies, where permitted, in conjunction with assessment, rehabilitation and treatment services. |
| Outpatient-Individual | Outpatient services provide a therapeutic environment that is designed to improve the functioning or prevent further deterioration of persons with mental health and/or substance abuse problems. These services are usually provided on a regularly scheduled basis by appointment, with arrangements made for non-scheduled visits during times of increased stress or crisis. |
| Outreach | Outreach services are provided through a formal program to both the community at large and to individuals. Community services include education, identification and linkage with high risk groups. Outreach services for individuals are designed to: encourage, educate, and engage prospective clients who show an indication of substance abuse and mental health problems or needs. Client enrollment is not included in outreach services. |
| Prevention | Prevention services are those involving strategies that preclude, forestall, or impede the development of substance abuse and mental health problems, and include increasing public awareness through information, education, and alternative-focused activities. These services may be directed either toward a Level II prevention target where the client has been identified, or at a Level I prevention target where the client is not identifiable. |
| Prevention/Intervention Day | This cost center includes school-based day services for children and adolescents for four (4) or more consecutive hours per day. For children with mental health problems, these services include school-based mental health services for children who have been identified by the school as having, or are at risk of developing, mental health problems. Services are individualized and may be provided in a self-contained classroom, a regular classroom, or as a component of a full service school. |
| Residential Level 1 | These licensed services provide structured, live-in, a non-hospital setting with supervision on a twenty-four (24) hours per day, seven (7) days per week basis. There is a nurse on duty in these facilities at all times.  For adult mental health, these services include group homes, which are for longer-term residents. These facilities offer nursing supervision provided by, at a minimum, licensed practical nurses on a twenty-four (24) hours per day, seven (7) days per week basis.  For children with serious emotional disturbances, Level I services are the most intensive and restrictive level of residential therapeutic intervention provided in a non-hospital or non-crisis support setting, including residential treatment centers. Medicaid Residential Treatment Centers (MRTC) and Residential Treatment Centers (RTC) are reported under this cost center. On-call medical care must be available for substance abuse programs.  For substance abuse, Level I services provides a range of assessment, treatment, rehabilitation, and ancillary services in an intensive therapeutic environment, with an emphasis on treatment, and may include formal school and adult education programs. |
| Residential Level 2 | These are licensed, structured rehabilitation-oriented group facilities that have twenty-four (24) hours per day, seven (7) days per week, supervision. Level II facilities are for persons who have significant deficits in independent living skills and need extensive support and supervision.  For children with serious emotional disturbances, Level II services are programs specifically designed for the purpose of providing intensive therapeutic behavioral and treatment interventions. This cost center includes services provided in Therapeutic Group Homes (TGH), Specialized Therapeutic Foster Homes (STFH) - Level I, and Therapeutic Foster Home (TFH) - Level I.  For substance abuse, Level II services provide a range of assessment, treatment, rehabilitation, and ancillary services in a less intensive therapeutic environment with an emphasis on rehabilitation, and may include formal school and adult educational programs. |
| Residential Level 3 | These are licensed facilities provide twenty-four (24) hours per day, seven (7) days per week supervised residential alternatives to persons who have developed a moderate functional capacity for independent living.  For adults with serious mental illness, this cost center consists of supervised apartments.  For children with serious emotional disturbances, Level III services are services specifically designed to provide sparse therapeutic behavioral and treatment interventions. This cost center provides services in Therapeutic Group Homes (TGH), Specialized Therapeutic Foster Homes (STFH)-Level I, and Therapeutic Foster Home (TFH) - Level I.  For substance abuse, Level III provides a range of assessment, rehabilitation, treatment and ancillary services on a long-term, continuing care basis where, depending upon the characteristics of the clients served, the emphasis is on rehabilitation or treatment. |
| Residential Level 4 | This type of facility may have less than twenty-four (24) hours per day, seven (7) days per week on-premise supervision. This is the least intensive level of residential care. It is primarily a support service and, as such, treatment services are not included in this cost center.  For adult mental health, Level IV includes satellite apartments, satellite group homes and therapeutic foster homes.  For children with serious emotional disturbances, Level IV services are the least intensive and restrictive levels of residential care provided in group or foster homes settings, therapeutic foster homes, and group care. |
| Respite Services | Respite care services are designed to sustain the family or other primary care giver by providing time-limited, temporary relief from the ongoing responsibility of care giving. Although the respite is for the caregiver, use the SSN of the client in question. |
| Substance Abuse Detoxification | Detoxification programs use medical and clinical procedures in a residential setting to assist adults, children and adolescents with substance abuse problems in their efforts to withdraw from the physiological and psychological effects of substance abuse. Residential detoxification and Addiction Receiving Facilities (ARFs) provide emergency screening, evaluation, short-term stabilization, and treatment in a secure environment. |
| Supported Employment | Supported employment services are community-based employment services in an integrated work setting, which provides regular contact with non-disabled co-workers or the public. A job coach provides long-term, ongoing support for as long as it is needed to enable the person served to maintain employment. |
| Supported Housing/Living | Supported housing/living services assist persons with substance abuse or psychiatric disabilities in the selection of housing of their choice. These services also provide the necessary services and supports to assure their continued successful living in the community and transitioning into the community.  For children with mental health problems, supported living is the process of assisting adolescents in arranging for housing and providing services to assure successful transition to living independently on their own or with roommates in the community. Services include training in independent living skills. |
| Treatment Accountability for Safer Communities (TASC) | TASC provides for identification, screening, court liaison, referral and tracking of persons in the criminal justice system with a history of drug abuse or addiction. |
| Incidental Expenses | This cost center provides for incidental expenses for items, such as clothing, medical care, educational needs, developmental services, FACT Team housing subsidies and pharmaceuticals and other approved costs. All incidental expenses must be included in the contract or must have prior written authorization from authorized department staff member. |
| Aftercare/Follow-up | Aftercare services, including but not limited to relapse prevention, are a vital part of recovery in every treatment level. Aftercare activities include client participation in daily activity functions, which were adversely affected by mental illness and/or substance abuse impairments. New directional goals such as vocational education or re-building relationships are often priorities. Relapse prevention issues are key in assisting the client's recognition of triggers and warning signs of regression. Aftercare services help families and pro-social support systems reinforce a healthy living environment. |
| Information & Referral | These services maintain information about resources in the community, link people who need assistance with appropriate service providers, and provide information about agencies and organizations that offer services. The information and referral process involves being readily available for contact by the individual; assisting the individual with determining which resources are needed; providing referral to appropriate resources; and following up to ensure the individual's needs have been met, if the individual agrees to such follow-up activities. |
| Behavioral Health Overlay Services | Medicaid funded behavioral health services provided as an overlay to residential group care. |
| FACT Team | These non-residential care services are available twenty-four (24) hours per day, seven (7) days per week, and include community-based treatment, rehabilitation and support services provided by a multidisciplinary team to persons with severe and persistent mental illness (SPMI) or to SPMI with co-occurring disorders. |
| Outpatient-Group | This cost center provides a therapeutic environment that is designed to improve the functioning or prevent further deterioration of persons with mental health and/or substance abuse problems. Outpatient-group services are usually provided on a regularly scheduled basis by appointment, with arrangements made for non-scheduled visits during times of increased stress or crisis. The group size limitations applicable to the Medicaid program shall apply to all outpatient services funded through a state substance abuse and mental health program contract. |
| Room & Board w/ Supervision, Level 2 | This cost center corresponds to cc [19] above for Residential Level II; same programs, units and data elements as cc [36] above for Room and Board with Supervision Level I. |
| Room & Board w/ Supervision, Level 3 | This cost center corresponds to cc [20] above for Residential Level III; same programs, units and data elements as cc [36] above for Room and Board with Supervision Level I. |
| Short-term Residential Treatment (SRT) | These individualized, stabilizing acute and immediately sub acute care services provide short and intermediate duration intensive mental health residential and habilitative services on a twenty-four (24) hour per day, seven (7) days per week basis. These services must meet the needs of individuals who are experiencing an acute or immediately sub acute crisis and who, in the absence of a suitable alternative, would require hospitalization. |
| Mental Health Clubhouse Services | This cost center provides structured, community-based services designed to strengthen and/or regain the client's interpersonal skills, provide psycho-social therapy toward rehabilitation, develop the environmental supports necessary to help the client thrive in the community and meet employment and other life goals and promote recovery from mental illness. Services are typically provided in a community-based program with trained staff and members working as teams to address the client's life goals and to perform the tasks necessary for the operations of the program. The emphasis is on a holistic approach focusing on the client's strengths and abilities while challenging the client to pursue those life goals. This service would include, but not limited to, clubhouses certified under the International Center for Clubhouse Development. |
| Intervention - Group | Intervention services focus on reducing risk factors generally associated with the progression of substance abuse and mental health problems. Intervention is accomplished through early identification of persons at risk, performing basic individual assessments, and providing supportive services that emphasize short-term counseling and referral. These services are targeted toward individuals and families. This cost center is used when reporting an individual's services which are provided in a group environment. Each individual within the group would have separate service event record to record group participation. |
| Aftercare - Group | This cost center includes services provided to individuals who have completed treatment in a licensable service component. Aftercare activities include client participation in daily activity functions which were adversely affected by mental illness or substance abuse impairments. New directional goals such as vocational education or re-building relationships are often priorities. Relapse prevention issues are key in assisting the client's recognition of triggers and warning signs of regression. Aftercare services help families and pro-social support systems reinforce a healthy living environment. This cost center is used when reporting an individual's services which are provided in a group environment. Each individual within the group would have separate service event record to record group participation. |
| Comprehensive Community Service Team - Individual | Comprehensive Community Service Team (CCST) services render assistance in identifying goals and making choices to promote resiliency and facilitate recovery to adults and children with mental illness. The services take place in either an outpatient or community based setting. For individuals with mental health problems, recovery is the personal process of overcoming the negative impact of psychiatric illness despite its continued presence. CCST services are intended to restore the individual's function and participation in the community. The services are designed to assist and guide individuals in reconnecting with society and rebuilding skills in identified roles in their environment. The focus is on the individual strengths and resources as well as their readiness and phase of recovery. A team approach to services will be used to guide and support the adults and children served with development of a recovery plan focusing on the areas of individual and family living, learning, working and socialization activities. Any therapy is brief and oriented toward skill building.  Services provided include Assessment, Case Management, Intensive Case Management, Supported Housing, Aftercare, Supported Employment , Outreach, Outpatient, In-home/On-site, Intervention, Information and Referral, Prevention, Prevention/Intervention and other transition and non-traditional support services as negotiated by the Department and the Provider. |
| Comprehensive Community Service Team - Group | Comprehensive Community Service Team (CCST) services render assistance in identifying goals and making choices to promote resiliency and facilitate recovery to adults and children with mental illness, the services take place in either an outpatient or community based setting. For individuals with mental health problems, recovery is the personal process of overcoming the negative impact of psychiatric illness despite its continued presence. CCST services are intended to restore the individual's function and participation in the community. The services are designed to assist and guide individuals in reconnecting with society and rebuilding skills in identified roles in their environment. The focus is on the individual strengths and resources as well as their readiness and phase of recovery. A team approach of services will be used to guide and support the adults and children served with development of a recovery plan focusing on the areas of individual and family living, learning, working and socialization activities. Any therapy is brief and oriented toward skill building.  Services provided include Assessment, Case Management, Intensive Case Management, Supported Housing, Aftercare, Supported Employment , Outreach, Outpatient, In-home/On-site, Intervention, Information and Referral, Prevention, Prevention/Intervention and other transition and non-traditional support services as negotiated by the Department and the Provider. |
| Substance Abuse Recovery Support Services - Group | These services are designed to strengthen and/or regain the person's skills, and to assist the person in developing the environmental support necessary to help him or her thrive in the community and meet life goals which promote recovery and resiliency. The focus is on person strengths and abilities while providing support for progress toward the person achieving recovery goals reflected in the person's screening, assessment, treatment plan, or discharge summary.  Services provided include substance abuse education, coordinating and planning in the treatment of medical or health problems, planning, coaching and guidance services that support education and employment objectives, family and marital and parenting guidance, life skills training and coaching, teaching anger and/or stress management coping skills, supportive counseling and other applicable services designed to facilitate recovery and resiliency that are approved by the Department. The services exclude twelve step programs including Narcotics Anonymous and Alcoholics Anonymous. This cost center is used when reporting an individual's services which are provided in a group environment. Each individual within the group would have separate service event record to record group participation. |
| Other | Other temporarily established |
